# The Diversity of Intermediate Filaments in Astrocytes

**DOI:** 10.3390/cells9071604

**Published:** 2020-07-02

**Authors:** Maja Potokar, Mitsuhiro Morita, Gerhard Wiche, Jernej Jorgačevski

**Affiliations:** 1 Laboratory of Neuroendocrinology – Molecular Cell Physiology, Institute of Pathophysiology, Faculty of Medicine, University of Ljubljana, 1000 Ljubljana, Slovenia; maja.potokar@mf.uni-lj.si; 2 Celica BIOMEDICAL, 1000 Ljubljana, Slovenia; gerhard.wiche@univie.ac.at; 3 Department of Biology, Kobe University Graduate School of Science, Kobe 657-8501, Japan; mmorita@boar.kobe-u.ac.jp; 4 Department of Biochemistry and Cell Biology, Max F. Perutz Laboratories, University of Vienna, 1030 Vienna, Austria

**Keywords:** astrocytes, intermediate filaments, GFAP, vimentin, nestin, synemin, plectin, cytolinker proteins, reactive gliosis

## Abstract

Despite the remarkable complexity of the individual neuron and of neuronal circuits, it has been clear for quite a while that, in order to understand the functioning of the brain, the contribution of other cell types in the brain have to be accounted for. Among glial cells, astrocytes have multiple roles in orchestrating neuronal functions. Their communication with neurons by exchanging signaling molecules and removing molecules from extracellular space takes place at several levels and is governed by different cellular processes, supported by multiple cellular structures, including the cytoskeleton. Intermediate filaments in astrocytes are emerging as important integrators of cellular processes. Astrocytes express five types of intermediate filaments: glial fibrillary acidic protein (GFAP); vimentin; nestin; synemin; lamins. Variability, interactions with different cellular structures and the particular roles of individual intermediate filaments in astrocytes have been studied extensively in the case of GFAP and vimentin, but far less attention has been given to nestin, synemin and lamins. Similarly, the interplay between different types of cytoskeleton and the interaction between the cytoskeleton and membranous structures, which is mediated by cytolinker proteins, are understudied in astrocytes. The present review summarizes the basic properties of astrocytic intermediate filaments and of other cytoskeletal macromolecules, such as cytolinker proteins, and describes the current knowledge of their roles in normal physiological and pathological conditions.

## 1. Introduction

The first observation of neuroglia, made by Rudolf Virchow in 1858, was rather dubious. He described it as a substance with a somewhat static role of holding together and giving form to the nervous parts. In the period of silver staining, pioneered by Camillo Golgi, glia was first recognized to possess proper cell characteristics and, before the turn of the 19th century, the most numerous cells of glia were named astrocytes [1]. The interest of the scientific community in astrocytes then slowed down for nearly a century, mainly because neurons were recognized as the single independent anatomical and physiological unit of the nervous system, capable of efficient communication over long distances [2]. Only when it became clear that astrocytes possess a specific form of calcium excitability, and that they are (in addition to microglia) implicated in inflammatory responses [3], did astrocytes recapture research attention in the scientific community [4,5,6]. Astrocytes were shown to be capable of propagating waves of calcium between the cytosols of neighboring cells, which, considering their abundance in the brain, was proposed to lead to an “extraneuronal pathway for rapid long-distance signal transmission within the central nervous system (CNS)” [5]. At the same time, Cornell-Bell and coworkers [5] proposed that calcium dynamics in astrocytes may influence neuronal activity in a bi-directional fashion. This new paradigm aroused the interest of several independent groups, which before long confirmed that astrocytes can respond to glutamatergic synaptic transmission [7] and vice versa—that calcium waves, initiated in astrocytes, can result in the modulation of neuronal activity [8,9]. Hence, the term tripartite synapse was coined, whereby astrocytes were recognized as partners of neurons that respond to synaptic activity and regulate synaptic transmission [10]. Research carried out soon after the identification of bi-directional communication between astrocytes and neurons suggested that one of the distinct pathways of astrocyte communication involves regulated exocytosis (i.e., the release of “gliotransmitters” by astrocytes) [9]. A quintessential step of exocytosis in astrocytes is vesicular transport [11], which precedes vesicle fusion and is governed not only by commonly associated cytoskeletal elements (microtubules and actin filaments) but also by intermediate filaments (IFs) [12,13]. Of course, the involvement of IFs in vesicular transport is distinct, because they are apolar, which prevents them being used as tracks, unlike microtubules and actin meshwork, which are utilized by specialized motor proteins that convert chemical energy into mechanical work [14].

Historically, the research on IFs in astrocytes did not start with vesicular transport. The first visualization of IFs overlaps to some extent with the discovery of astrocytes, because Golgi’s silver staining and its variations (notably Ramón and Cajal’s own astrocyte-specific gold sublimate stain) target IFs among other cellular components [15]. Throughout the 20th century, a series of IF-related discoveries were made, mainly in the field of keratins, which coincided with the development of new methodologies and methods for the preparation of biological specimens (for an interesting review, see [16]). One of the greatest challenges in the early stages of IF research was the classification of different IF members in a common family. In contrast to highly conserved actin filaments and microtubules, IFs derive from approximately 70 different genes, and the diversity of expressed proteins is further increased by multiple splice variants of the same genes [17,18]. In addition, although IFs are still commonly used as cell type markers, many cell types express more than one type of IF, and the expression of individual IFs frequently also depends on the physiological state of the cell. For example, glial fibrillary acidic protein (GFAP) was considered to be a reliable marker of glial and astrocyte cell identity, yet current investigations have shown that GFAP is not an absolute marker of all non-reactive astrocytes because it is often not immunohistochemically detectable in astrocytes of healthy CNS tissue remote from CNS lesions [19]. Be that as it may, starting with the molecular characterization of α-keratin in the early 1960s [20], ultrastructural analysis provided the first repeating pattern of fibers in different cell types; the average diameter of these fibers was measured at ~10 nm [21,22,23]. This diameter is intermediate between that of microfilaments (~6 nm) and microtubules (~24 nm), which earned this group of filaments its name [23]. At the time, various IFs were given a number of different names and were frequently misidentified as microtubules [16]. In the 1970s and 1980s, several types of IFs had been isolated, owing to their conspicuous insolubility, which, combined with the development of molecular tools (including antibodies against individual IFs), facilitated the research on IF structure and intracellular localization [24,25,26,27]. Systematic analysis of the molecular organization of IFs revealed a typical tripartite structure, a globular N terminus (head) and C terminus, connected by a central α-helical domain (the “rod”) with a number of coiled-coil segments of conserved size [28]. Based on their amino acid sequence, protein structure and tissue-specific expression patterns, IFs were initially classified into five groups [29]; however, after the discovery of nestin, Group VI was added to the classification [30]. It was in 1971 that the first IF was identified in astrocytes. A well-known characteristic of astrocytes, i.e., their ability to form fibers under a variety of pathological conditions (fibrous gliosis), together with the acidic properties of the new isolated protein, were merged into the name glial fibrillary acidic protein [3]. In the years that followed, astrocytes were also shown to express vimentin, nestin, synemin and lamins, which intricately interact with each other as well as with other filament types.

## 2. Glial Fibrillary Acidic Protein and Vimentin

### 2.1. Structure and Expression of Glial Fibrillary Acidic Protein in Astrocytes

GFAP forms characteristic fibres in astrocytes and was first isolated from sections of elder human brain that showed severe fibrous gliosis [3]. A substantial amount of research in the five decades since the discovery of GFAP has resulted in extensive knowledge of its properties and functions. GFAP is the principal astrocyte IF protein and is widely used as a diagnostic marker for astrocyte-derived human neoplasms and as a marker of astrocytes and astrocyte precursors [31,32]. Despite high specificity for astrocytes, GFAP is also expressed by neural stem cells and, albeit rarely, in non-glial cells [33,34,35]. Therefore, it was suggested that the proper detection of astrocytes should combine the identification of different astrocyte markers, such as ALDH1L1 [36]. Ten splicing variants of GFAP mRNA have been described so far, as shown in Table 1 (reviewed in [18,37]), ranging in approximate size between 38 and 50 kDa [38]. GFAP is a Type III IF [39] and, similar to other type III IFs, is able to form filaments without a binding partner, although such homopolymers show atypical organization and tend to cluster, as observed in mouse CNS [40,41].

The aberrant appearance of IFs is otherwise typical for a range of epithelial, muscle and neuronal disorders, including gliomas and Alexander disease. GFAP is a standard marker of more differentiated astrocytoma (i.e., a type of glioma with astrocyte features). However, a thorough analysis of the reports focusing on GFAP expression in patients with astrocytoma did not confirm the correlation between general GFAP expression and astrocytoma malignancy [60]. Instead, van Bodegraven and coworkers proposed that astrocytoma malignancy is well reflected by the ratio of GFAP isoforms GFAPδ and GFAPα (a canonical GFAP isoform), which could improve the accuracy of assessing the differentiation state of this type of glioma [60]. GFAPδ is an endogenous GFAP isoform and is typically expressed at low levels (~10%) [61]. In astrocytoma cells, the silencing of the GFAPα isoform increased the GFAPδ/GFAPα transcript level ratio, as well as the expression of the extracellular matrix protein laminin. Simultaneously, the downregulation of plectin mRNA and protein levels was observed, which together resulted in the decreased mobility of cells [62]. Natural occurrence of GFAP isoforms other than GFAPα, such as GFAPδ, implies that GFAP filaments can accommodate a small proportion of other residues, including those with aggregation-prone mutations [61]. Alexander disease is intrinsically linked to heterozygous mutations of GFAP gene [41,63]. Rosenthal fibers are composed of numerous proteins, including several IFs, such as GFAP, vimentin, synemin and the cytolinker, plectin [64,65,66]. Vimentin and nestin are GFAP co-polymerization partners that form heteropolymers in astrocytes [41,67], whereas GFAP merely associates with synemin [68].

Similar to other IFs, GFAP also shows a developmental pattern of expression. Its expression in rat cortex gradually increases from embryonal to adult stage, showing an inverse trend compared with other IFs (vimentin, nestin, synemin) [69]. The expression of GFAP in astrocytes is further enhanced in pathological conditions, such as neurotrauma and neurodegenerative disorders [32], as reviewed in Section 2.1. So far, the function of GFAP has been linked to a variety of processes implicated in the signaling and structural properties of astrocytes, ranging from resistance to mechanical stress, mitosis, the anchoring of transporters, the motility of cells, the mobility of vesicles and resistance to oxidative and electrophilic stress, as shown in Table 1 [13,32,37,42,43,70]. Functional studies of GFAP in astrocytes are in many cases linked and interpreted in combination with its polymerizing partner vimentin.

### 2.2. Structure and Expression of Vimentin in Astrocytes

Vimentin is the most common co-polymerization partner of GFAP and is considered as the principal IF, at least in adult astrocytes [71]. The discovery of vimentin, which is also a Type III IF, dates back to 1978, when it was isolated from a murine embryonic fibroblast cell line, as shown in Table 1 [46]. Reports of astrocytes positive for vimentin followed soon after [72,73,74,75].

Vimentin synthesis begins early during mammalian embryogenesis, and assembled vimentin filaments were observed somewhere around days E7–E11 of mouse embryogenesis, depending on the cell type [75]. Vimentin expression in astrocytes decreases with development and may completely cease in adult CNS or remain expressed at detectable levels. For example, Bergmann glia, Müller glia, radial glia and a subset of cortical astrocytes continue to express vimentin (together with GFAP) in the adult stage, suggesting a functional role for vimentin in these cells [69,75,76]. Generally, the tendency of vimentin expression to cease is reversed in different neurological conditions [48]; its increased expression in reactive astrocytes in conjunction with GFAP is reviewed in Section 2.1.

**Figure 1:**
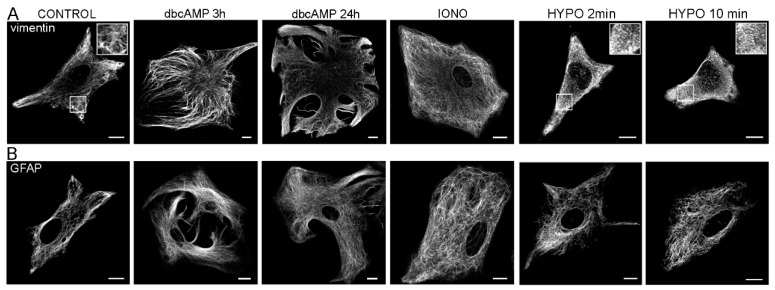
Cellular distribution of GFAP and vimentin cytoskeleton in primary rat astrocytes in normal conditions and in conditions that are typically present in pathological states. Astrocytes treated with dbcAMP (N 6,2′-O -dibutyryladenosine 3′:5′ cyclic monophosphate), a membrane-permeable analogue of cAMP, mimic general reactive gliosis. Hypotonic stimulation, on the other hand, leads readily to astrocyte swelling, which is a part of the cytotoxic or cellular edema response. Changes in intracellular arrangement of vimentin (**A**) and GFAP (**B**) filaments are evident in reactive astrocytes (after cAMP stimulation) and after hypotonic stimulation (HYPO), as revealed by immunolabeling. Note also the stellated morphology of astrocytes after the increase in cAMP. Hypotonic treatment triggered depolymerization of vimentin filaments—selected areas (white squares) are magnified (2×)—in insets Bars: 10 µm. Modified with permission from [84] (Regulation of AQP4 Surface Expression via Vesicle Mobility in Astrocytes, GLIA, Copyright© 2013 Wiley Periodicals, Inc., (Hoboken, NJ, USA)).

Vimentin shares high sequence homology throughout vertebrates [77]. Similar to GFAP, it has the ability to assemble into homopolymers or form heteropolymers with its co-polymerization partners—these are GFAP, nestin, and synemin in the case of astrocytes [41,68]. Vimentin filaments are highly dynamic structures. Similar to other IFs, their structure and assembly is regulated by phosphorylation [41,78,79,80], influencing several cellular functions. As demonstrated in different cell types, vimentin acts as an organizer of numerous crucial proteins involved in attachment, migration and cell signaling [77]. Similarly, in astrocytes, vimentin has been demonstrated to influence mechanical stability and the morphology of cells, cell motility, vesicle trafficking, the guiding of progenitors along the glial scar, cell division and signaling where phosphorylation reactions play a significant part, as shown in Table 1 [13,41,44,49,81]. Phosphorylation also regulates the interactions of vimentin with 14-3-3 proteins, as demonstrated by the application of the phosphatase inhibitor calyculin A [82]. The 14-3-3 dimeric phosphoserine/threonine-binding molecules are found in all eukaryotic organisms and participate in developmental processes, signal transduction, checkpoint controls, nutrient sensing, and cell survival pathways [83]. The sequestering of 14-3-3 molecules by phosphorylated vimentin is predicted to limit its availability to other target proteins, thereby affecting intracellular signaling processes that require pathways linked to 14-3-3 [82]. The phosphorylation-induced depolymerization of vimentin filaments by calyculin A in astrocytes was demonstrated to severely reduce vesicle trafficking [13]. Similarly, the absence of vimentin expression in astrocytes devoid of IFs (*GFAP^−/−^Vim^−/−^* astrocytes) attenuates the displacement of vesicles, supporting the hypothesis that IFs are required for long-range directional vesicle mobility by acting as a three-dimensional lattice [13]. A hypothesis has been proposed that the upregulation of IFs in pathological states may alter the function of astrocytes by deregulating the vesicle trafficking of vesicles carrying peptide, transporters and vesicles in endosomal/lysosomal pathways [11,12,43]. Altered vesicle trafficking is also related to the redistribution of IFs in conditions that are typically present in such states, as shown in Figure 1. 

### 2.3. Reactive Gliosis

As a consequence of any insult to the CNS (e.g., trauma, stroke or ischaemia), astrocytes respond by changing their phenotype and gene expression. Hallmarks of this response, which is referred to as reactive gliosis (also astrogliosis), are hypertrophy, proliferation and metabolic changes, which have a multifaceted impact on pathological processes. The progression of neurodegenerative diseases, including Alzheimer’s disease and amyotrophic lateral sclerosis, is associated with the accumulation of reactive astrocytes producing toxic substances, such as reactive oxygen species and matrix metalloproteases [85,86], whereas recovery from brain injuries is exacerbated by the ablation of reactive astrocytes [87,88]. The production of extracellular matrix and factors promoting synapse formation or pruning by reactive astrocytes is a determinant of prognosis for neuropathological conditions, including post-traumatic epilepsy [89,90].

**Figure 2:**
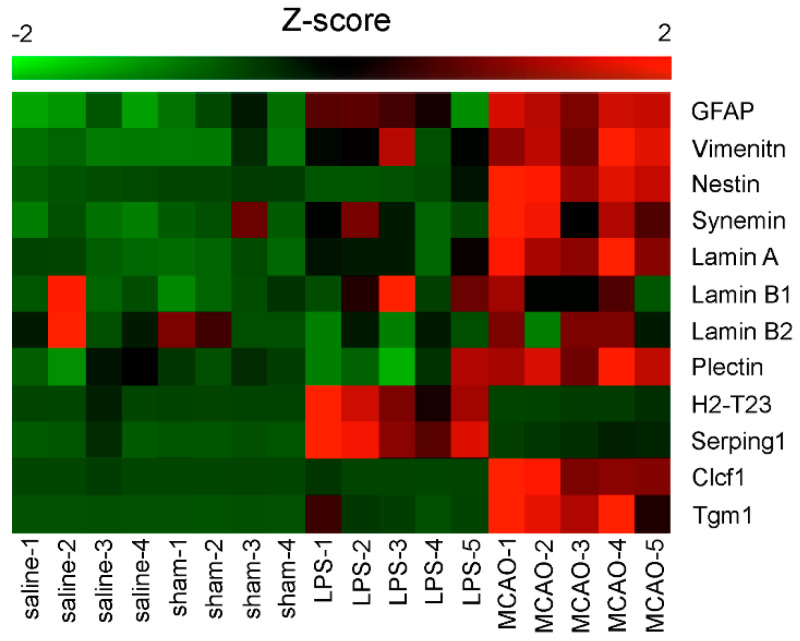
Expression profile of intermediate filament and cytolinker genes in A1 and A2 reactive astrocytes. Microarray data provided in [98] are converted to Z scores and expressed as a heatmap. Saline-1 to -4 are controls of LPS treatments (A1 reactive astrocytes) and sham-1 to -4 are controls of MCAO (A2 reactive astrocytes), where LPS is *Escherichia coli* endotoxin O55:B55 and MCAO stands for transient ischaemia induced by occluding the middle cerebral artery. H2-T23 and Serping 1 are representative genes highly and selectively expressed in the A1 astrocyte subtype, whereas Clcf1 and Tgm 1 are specific for the A2 astrocyte subtype.

Reactive astrocytes are derived not only from astrocytes but apparently also from non-astrocytic cells, such as neural stem cells or oligodendrocyte progenitor cells [91,92,93]. However, the significance of reactive astrocytes derived from neuron-glial antigen 2 (NG2) expressing glia progenitors 2 is controversial, because another line of evidence shows that a subset of astrocytes deriving from NG2 expressing glia progenitors is generated only in embryonic or fetal tissue [94]. Thus, reactive astrocyte populations may consist of multiple cell types that are functionally diverse, and the selective detection and manipulation of these subpopulations is proposed to have clinical relevance in a number of conditions related to brain disorders. In vitro studies of reactive astrocytes have demonstrated competitive regulations of astrocyte functions by pro-inflammatory cytokines and growth factors and suggested the existence of diverse types of reactive astrocytes [95,96]. In agreement, transcriptome analysis of reactive astrocytes induced by inflammation or brain injury showed distinct gene expression profiles [97], and these reactive astrocytes were designated as A1 and A2 subtypes in subsequent publication [98]. The distinct expression of IF genes is summarized in Figure 2, which is based on the transcriptome databased related to the initial study [97]. Interestingly, GFAP is upregulated in both the A1 and A2 reactive astrocytes, whereas vimentin expression is more prominent, and nestin and plectin appear to be exclusively upregulated in the A2 subtype. It is not clear if microarray data included plectin rodless variants. The expression levels of these variants in the mouse brain are approximately 20-times lower compared with the full-length counterparts [99]; however, it is not clear how the reactivation of astrocytes affects their expression levels.

Mouse models lacking IFs are a powerful tool for studying the formation and role of IFs in normal or reactive astrocytes in vivo and in astrocyte cultures [41]. The involvement of IFs in the functioning of reactive astrocytes has been studied extensively using *GFAP^−/−^* and/or *Vim^−/−^* mice. Reactive gliosis in *GFAP^−/−^Vim^−/−^* mice is generally less pronounced, which is detrimental for the initial spread of the injury; however, it is beneficial for the later regenerative phase [100,101]. Furthermore, the distinct roles of IFs in the A1 and A2 subtypes of reactive astrocytes [98] are suggested in studies of reactive gliosis in various pathological processes. Differences in the parameters of the affected area after brain injury, where predominantly the A2 subtype of reactive astrocytes accumulates, were observed only in the *GFAP^−/−^Vim^−/−^* mice. For example, glial scar formation after stab injury is reduced in the double knockout mice but not in the *GFAP^−/−^* mice [48,101]. Similarly, the infarct size after permanent occlusion of the middle cerebral artery and the lesion size after spinal cord injury are larger in the *GFAP^−/−^Vim^−/−^* mice [102,103]. Moreover, after brain injury, reactive astrocytes possess fewer and shorter processes in the double knockout mice. These results suggest that vimentin is crucial for neuroprotective A2 astrocytes, whereas GFAP deficiency can be compensated by the upregulation of vimentin and crosslinking capacity of plectin. In contrast, A1 reactive astrocytes, which accumulate in animal models of brain inflammation, were affected in the *GFAP^−/−^* mice, presumably due to the lower level of vimentin and plectin upregulation. Reactive astrocytes in an Alzheimer’s disease model were identified as the A1 subtype by gene profile analysis [98], and the reaction of astrocytes to β-amyloid was suppressed in the *GFAP^−/−^* mice [104]. The progression of neurodegeneration in a brain autoimmune disease, called experimental autoimmune encephalomyelitis, was accelerated in the *GFAP^−/−^* mice, reflecting the incomplete formation of the glial border surrounding a lesion [105]. The accumulation of reactive astrocytes after cerebral bacterial infection was suppressed in the *GFAP^−/−^* mice, resulting in an increase in immune cell infiltration [106]. This line of evidence suggests that the IFs of A1 reactive astrocytes are largely composed of GFAP alone, and the ablation of GFAP is sufficient for suppressing hypertrophic morphology.

## 3. Nestin

### 3.1. Structure of Nestin

Nestin is one of the earliest expressed IFs during brain development and is still regarded as a neural progenitor cell (NPC) marker. The discovery of nestin coincides with a search for suitable markers of major cell types in the developing nervous system. Hockfield and McKay observed that antibody “Rat-401” identified proliferating cells in the early neural tube of rats [50]. The epitope recognized by Rat-401 was shown to belong to a protein that was encoded by a gene specifically expressed in neuroepithelial stem cells and thus named nestin [107]. True to its name, nestin expression was shown to be downregulated after the differentiation of NPCs into neurons or glial cells [108,109]. Nonetheless, ensuing studies revealed that nestin expression is more promiscuous than suggested initially; in addition to NPCs, other proliferative cell types, such as myoblasts and cancer cells (nestin is a useful marker of high-grade gliomas [110]) and even a subpopulation of microglia, have been reported to express nestin [111,112,113,114,115]. Moreover, together with vimentin and synemin, nestin is also expressed in immature astrocytes [69,116]. The expression of nestin depends on the ubiquitin proteasome system, similar to other IFs [117], and is restarted in reactive astrocytes [41,118,119], as further elaborated in Section 2.1.

Nestin can form homodimers and homotetramers in vitro but cannot form IFs per se (similar to synemin). In astrocytes, nestin can form heteropolymeric filaments with either vimentin or GFAP as obligatory partners [41,48,120,121]. In accordance, different GFAP isoforms were shown to control intermediate filament network dynamics, including those of nestin [122]. The intricate collaboration of different types of IFs in filament formation is governed by the structure of individual IFs and the nature of their interaction. In this respect, the interaction between the acidic α-helical rods and the basic N-terminal head domain is critical for the formation of IFs. In the case of keratins and vimentin, the complete removal of the head domain was shown to impair IF assembly beyond tetramers [123,124]. In contrast to the extremely long tail domain (more than 1300 amino acids in human and 1500 in rat nestin [30,125]), the nestin head domain consists of only six amino acids. For comparison, the head domain of vimentin and GFAP is composed of 82 and 48 amino acids, respectively [126]. Therefore, the relatively small head domain of nestin appears to be the key structure that precludes nestin assembling in filaments on its own.

Upon its identification, nestin was recognized to possess a unique structure and thus became the prototype for a new IF protein group: Type VI [107]. Nestin shares a common structure with the other IFs (as recently reviewed in detail in [127]) and exhibits gene structure and protein similarities, especially with neurofilaments. Consequently, it was proposed that nestin should be placed, together with neurofilaments, in Group IV IFs [125]; however, the unique α-helical region and the presence of a third intron in the nestin gene were finally decisive for nestin to be classified in a distinct group— Group VI. Nestin did not occupy Group VI of IFs alone for long. The sequencing of synemin [54], as well as discoveries of tanabin (IF expressed in amphibians [128]) and transitin (IF expressed in birds [129]), eventually joined nestin in Group VI IFs [130].

### 3.2. Expression and Function of Nestin in Astrocytes

Our knowledge of the functions of nestin in astrocytes is still very limited. Functions of nestin may also be linked to its localization. The pattern of nestin distribution in astrocytes shows that nestin protein and nestin mRNA are more prominently localized in cell protrusions than GFAP and vimentin. Nestin’s extensive localization in astrocyte protrusions may have an important effect on the reorganization of astrocyte morphology during CNS development and maintenance [131].

The upregulation of IFs (GFAP, vimentin) in astrocytes has a neuroprotective role in the initial phase of brain injury, yet in the regenerative phases, it might also exhibit certain negative effects [100,101]. Accordingly, downregulation of the expression of IFs at the later phases of brain injury may improve the neurosupportive properties of astrocytes. Clomipramine, which is classified as a tricyclic antidepressant, was shown to be a promising candidate for this task. Clomipramine did not affect astrocyte resilience to oxidative stress; it decreased the protein levels of GFAP, vimentin and nestin and promoted the attachment and survival of neurons co-cultured with astrocytes [132]. However, nestin is not relevant solely in the reactive astrocytes of injured brains, because nestin is also apparently expressed in a subset of astrocytes in the unchallenged hippocampus. Moreover, the expression of nestin in astrocytes negatively regulates adult neurogenesis; this effect is mediated by notch signaling, which is crucial in the differentiation stages of stem cells [51,133]. Nestin-containing cells, including immature astrocytes, produce Notch1 mRNA [134]. Notch1 is one of the three Notch receptors involved in the elaborate signaling system that plays many important roles in development and has been shown to promote the proliferation and differentiation of reactive astrocytes, in particular within the subventricular zone [135,136]. The absence of nestin in astrocytes likely impairs notch signaling by affecting trafficking as well as the exo- and endocytosis of vesicles containing the Notch ligand, Jagged-1 [137,138]. As a functional consequence of impaired notch signalling, nestin-deficient (Nes^−/−^) mice showed improved (reversal place) learning as well as memory extinction on account of facilitated forgetting [51,139].

It has been proposed that, in progenitor cells, nestin, through promoting the disassembly of vimentin filaments, has an important effect on the trafficking and delivery of IF proteins and other cellular elements to daughter cells during cell division [140]. Cytoplasmic IFs, including nestin, are involved in a dynamic turnover, which depends on intracellular retrograde and anterograde transport, mediated by actin and microtubule meshworks, yet they also influence cell mobility. Wound-induced astrocyte polarization induces the protein kinase C-dependent inhibition of dynein-dependent retrograde transport, which promotes IF transport directed towards the cell front [141]. In turn, IFs (vimentin, GFAP and nestin), in combination with the cytolinker plectin, govern the collective migration of astrocytes by participating in the dynamics of the acto–myosin network [44]. The genetic ablation of nestin alone is sufficient to cause a significant decrease in astrocyte migration [51]. The crosstalk between the acto–myosin network and microtubules during the mobility of astrocytes is mediated by adenomatous polyposis coli (APC), which is known to be a tumor suppressor, regulating cell differentiation [142]. These results validate the active participation of IFs in astrocytes in adult neurogenesis, CNS regulation and plasticity.

## 4. Synemin

### 4.1. Structure and Expression of Synemin

Synemin, a high-molecular-weight polypeptide, was first isolated from smooth and skeletal muscle from chicken [52]. Later, it was discovered that human smooth and striated muscles express two synemin isoforms—α synemin and β synemin (desmuslin)—that are encoded by a single gene [53,54]. On the other hand, mouse skeletal muscles express three isoforms: H and M isoforms, which are similar in size to human α (180 kDa) and β (150 kDa) synemin, respectively, and a much smaller L isoform (41 kDa) [143].

The expression of synemin in astrocytes was initially shown for rat brain [69] and subsequently demonstrated by several other studies. Its expression follows a developmental pattern, whereby subpopulations of astrocytes in cerebral cortices of rat embryos, new-born and postnatal pups were shown to express synemin, whereas the cortices of adult (>60 days old) rats were devoid of synemin [69]. A similar developmental pattern of synemin expression was detected in mouse primary cultures of GFAP and vimentin positive glial cells, prepared from embryonal (E16) brain and dorsal root ganglia, where synemin also showed a decrease in expression during development [144]. Similarly, adult astrocytes of rabbit spinal cord also lack synemin [145]. Interestingly, however, astrocytes in adult bovine and rabbit optic nerves and astrocytes in adult rat retina express at least low levels of synemin [145,146]. Beside the CNS, synemin was also found in rabbit non-myelin-forming Schwann cells of the peripheral nervous system [145].

In contrast to muscle cells [143], astrocytes and other glial cells (ependymal cells, Schwann cells), along with neural precursors, express H and M synemin isoforms. There are no reports that astrocytes also express the third isoform, L synemin. Although neurons in rat cerebral cortices are reported to be apparently devoid of synemin [69], L synemin was detected in the neurons and ependymal cells of adult mice, as well as in neurons differentiated from mouse pluripotent embryonal stem cells [144,147]. In addition, human astrocytic tumors contain the H and M synemin orthologues α synemin and β synemin [55].

### 4.2. Subcellular Localization of Synemin

Synemin was found closely associated with desmin and vimentin when discovered in myotubes, suggesting that it either interacts with other IFs or co-polymerizes with them [52]. Later, both assumptions proved accurate. In mammalian muscle tissue, both α- and β synemin were shown to be incorporated with desmin into heteropolymeric IFs [54]. In addition, experiments in mouse astrocytes demonstrated that α synemin interacts with other IFs, namely with GFAP and vimentin filaments [68]. It appears that synemin interacts with GFAP as an IF-associated protein, whereas with vimentin, it acts as a polymerization partner that may function as an adaptor protein to enable synemin incorporation into GFAP filaments [68]. It was proposed that synemin functions as a key crosslinking protein that connects different cytoskeletal components [120,148], but thus far this theory has not been properly tested in astrocytes. Synemin’s inability to polymerize into filaments without a binding partner may be due to its short (10 amino acids) head domain, which is much smaller compared with the roughly ten-times longer head domains of vimentin or desmin [54]. The ability of synemin to interact with other proteins originates from its long C-terminal tail domain that can link heteropolymeric IFs to other cytoplasmic components, such as vinculin and α-actinin, in striated muscle cells [120,148].

Knowledge about the interactions of synemin with different cytoskeleton proteins, along with its subcellular distribution in astrocytes, is limited. The small number of studies that have dealt with this issue have shown that the subcellular localization of synemin in astrocytes is heterogeneous. Synemin can be found along the dense IF network around the nucleus, yet it is also present along dispersed fibers in other cell regions [68]. Similarly, little is known about the detailed association with other cytoskeletal proteins in astrocytes. In cultured astrocytes from mouse, where IF protein composition resembles that of reactive astrocytes, synemin apparently does not co-localize with IF network-linked structures rich in α-actinin or vinculin [68]. Nevertheless, synemin’s presence in the ruffled parts of the plasmalemma of human astrocytoma cells, which are rich in α-actinin, opens the possibility that, in tumors, synemin may interact with cell membranes and possibly play a role in cell motility [55].

### 4.3. Synemin in Reactive Astrocytes

When astrocytes become reactive, they start to express synemin, as was shown after neurotrauma in mice and in human reactive astrocytes, where the expression of synemin was confirmed around ischaemic lesions or epileptic foci [55]. Similar to the CNS, the upregulation of synemin has been reported in retinal astrocytes in adult rats [146]. Therefore, the expression of synemin in reactive astrocytes may be another promising marker for reactive gliosis in adults [68]. Reactive astrocytes express synemin, which is predominately, but not exclusively, present in GFAP-positive astrocytes [68]. In addition to reactive astrocytes in mice, synemin’s presence has been immunohistochemically documented in the human tissue of patients with Alexander disease [65]. Synemin was also detected in many among the GFAP-positive reactive astrocytes in Rosenthal fibers, where it aggregated with GFAP, the small stress proteins, HSP27 and αB-crystallin [65].

Further studies are needed to elucidate the meaning of specific synemin interactions with other cytoskeleton proteins. With understanding of its developmentally regulated, as well as cell type-specific expression pattern, including its expression in injured neuronal tissue (reactive astrocytes), we will get a deeper insight into synemin’s physiological (and pathological) functions.

## 5. Lamins

Unlike the other IFs, which are predominantly cytoplasmic, lamins form a scaffold in the nucleoplasm, primarily at the nuclear periphery. In vertebrate cells, lamins were initially described by Fawcett [149], who suggested (in 1966) that apart from the structural support, other functions of “fibrous lamina” should be considered. This conclusion was drawn from the comparison of nuclear size and the thickness/presence of this layer in cells of vertebrates and invertebrates. Today, lamins are regarded as the only IFs that are universally expressed in metazoans [150]; in the 1960s, there were only a few reports on the nuclear lamina. Nonetheless, Fawcett’s suggestion proved to be appropriate, because, analogous to other IFs, lamins are responsible not only for maintaining the structural integrity of the nucleus but they also participate in a multitude of other cellular functions, including higher-order genome organization, the regulation of chromatin, DNA replication and repair, and nuclear assembly/disassembly [151,152]. Ten years after their fibrous nature was described in vertebrate cells, Aaronson and Blobel [153] reported that nuclear lamina contains three major structurally related polypeptides, which were later named lamins A, B and C [154]. The ensuing biochemical characterization of lamins, isolated from baby hamster kidney cells and cDNA cloning (human T cells), have classified nuclear lamins as Type V IFs [155,156].

In mammalian somatic cells, four major lamin isoforms are expressed: lamins A and C, which are classified into A-type lamins (encoded by the *LMNA* gene) and lamins B1 and B2, which represent B-type lamins (encoded by the *LMNB1* and *LMNB2* genes, respectively) [151]. In glial cells, lamins (intranuclear fibrils) were first reported in the ventral nerve cord of the leech *Hirudo medicinalis* [157]. In vertebrates, biochemical and immunohistochemical studies confirmed the presence of lamins in the brain in the 1980s [158,159]. Soon after, their expression was also identified in all glial (and neuronal) cells in rat CNS [160]. However, not all lamin isoforms are expressed simultaneously during development. Lamins A and C are largely lacking in the developing embryo (mouse) brain (as well as from several types of epithelial tissues), because their expression in the brain occurs only several days after birth [161,162]. Conversely, all embryonic cells that are lamin A and C negative are lamin B positive [161], and lamin B1 levels apparently modulate the differentiation of murine neural stem cells (NSCs) into neurons (NSCs expressing high levels of lamin B1) and astroglial-like cells (NSCs expressing low levels of lamin B1) [163]. In adult animals, lamins A and C are found in similar amounts in most tissues, aside from the brain (with the exception of endothelial and meningeal cells). This disproportion has been linked to the expression of a brain-specific microRNA, miR-9; glial cells and neurons of mice express high levels of miR-9, which results in the downregulation of prelamin and lamin A, but not lamin C expression [164]. In agreement, a recent report corroborated that adult rat astrocytes showed immunoreactivity for lamins B1, B2 and C, but not for lamin A [165]; however, some of the lamin A-positive astrocytes that did not express GFAP (marker used by the authors) may have been missed in this report.

Many important roles of lamins in the structural organization of the nucleus and chromatin, as well as genome regulation, have been discovered in the past two decades (reviewed in [151,152]). Some of their functions are exerted by the direct binding of lamins to different replication and transcription factors (e.g., proliferating cell nuclear antigen [57]), whereas other functions are indirect and are mediated by lamin-binding proteins, such as emrin [166]. As the major component of lamina-genome interaction, lamins most likely are also involved in the control of gene expression programs during the lineage committed differentiation of neural precursor cells into astrocytes [167]. Noteworthy, some of the lamin-binding proteins also connect lamins to the cytoplasm, including to the cytoskeleton. The interaction to the cytoskeleton involves the linker of the nucleoskeleton and cytoskeleton (LINC) complex, which connects lamins to the actin and intermediate filament cytoskeletons via direct or plectin-mediated interactions [168,169].

### Lamins and Diseases

The research interest in lamins gained traction in the 1990s, when mutations in genes encoding proteins of the nuclear lamina, especially lamins, were shown to cause different pathologies [170,171]. Since then, around 15 diseases, which are also known under the name laminopathies, were linked to mutations in *Lamin* genes in humans [172]. Most of the laminopathies, such as lipodystrophy, cardiac dystrophy, muscular dystrophy and skin or bone defects, affect tissues of mesodermal origin [173]. Charcot-Marie-Tooth (CMT) disease encompasses a heterogeneous group of genetic disorders, which is characterized by the loss of peripheral nerve myelination, affecting both the motor and sensory nerves. Certain mutations in *LMNA* can cause CMT [174], but the CNS in LMNA knockout mice is unaffected [175]. Nonetheless, clinical features typical of a CMT neuropathy have been observed in patients with Fragile X-associated tremor/ataxia syndrome (FXTAS) [176]. FXTAS is a neurodegenerative disorder with heterogeneous clinical presentation, initiated by a CGG repeat expansion in the *fragile X mental retardation 1* gene, which manifests as intranuclear inclusions in neurons and astrocytes [177]. The presence of lamin A and C in the neuronal and astrocytic intranuclear inclusions of FXTAS resembles the histopathology of some disease-forming *LMNA* mutations, which suggests a functional connection between FXTAS and CMT-type neuropathies [178,179]. Duchenne muscular dystrophy is another muscular disorder in which (CNS-related) cognitive disturbances and neuropsychiatric symptoms are prevalent. In this case, they are attributed, among others, to mutation in the Dp71 variant protein, which is expressed in neurons and glial cells [180]. Interestingly, the expression of Dp71, which co-localizes with lamin B in healthy astrocytes, is decreased in glioblastoma cells and localized in the cytoplasm, whereas the expression of lamin B is increased [181]. Lamins A and C, on the other hand, play a prominent role in the transport of neurofibromin to the nucleus in astrocytes and glioblastoma cells [58]. Neurofibromin is a tumor suppressor that regulates RAS signaling in the cytoplasm, and hence also its downstream mediators, PI3K (phosphatidylinositol 3-kinase)/mTOR (mammalian target of rapamycin) [182]. mTOR plays a crucial role in regulating autophagy, which has been observed in astrocytes in conditions mimicking those present at ischaemic stroke and was accompanied by lamin A cleavage [183].

Lamins in astrocytes have been proposed to also play a role in viral infections (lamins A and C may promote virus egress [184]), amyotrophic lateral sclerosis (GFAP-expressing astrocytes in lumbar spinal cord express nuclear p16^INK4a^, which is typically accompanied by the loss of lamin B1 [185]) and even during chronic alcohol exposure (the decreased expression and redistribution of lamins A and C affect nucleocytoplasmic transport [59]). Further investigation is warranted to gain full insights into lamin-related functions in astrocytes and, especially, to define the clinical significance of the astrocytic phenotypes in certain laminopathies.

## 6. Cytolinkers and Related Proteins

### 6.1. Structure and Expression in Astrocytes

In eukaryotic cells, the three main types of cytoskeletal filaments form highly organized networks, which are interconnected by crosslinking proteins that also ensure their anchorage to junctional complexes. The crosstalk between different cytoskeletal filament systems, mediated by crosslinking proteins, is essential for a variety of biological processes in the which synchronous response of more than one filament type is required, such as in cell migration. Giant cytolinkers of the plakin/spectraplakin family of proteins represent some of the most important cytoskeletal linker elements [186,187]. Of the nine family members discovered to date, seven have been identified in mammals: bullous pemphigoid antigen 1 (BPAG1; also known as dystonin), microtubule actin crosslinking factor 1 (MACF1; also called ACF7 [188], trabeculin [189] and macrophin [190]), plectin, desmoplakin, envoplakin, periplakin and epiplakin [191]. With the exception of MACF1 and epiplakin, mammalian plakins share a plakin domain (consisting of two stretches of spectrin repeats separated by a Src-Homology 3 [SH3] domain) that mediates protein–protein interactions. In addition, individual plakins comprise distinct combinations of various domains, including an actin binding domain (ABD), coiled-coil rod domains of varied lengths, spectrin repeat-containing rod domains, different numbers of plectin/plakin-repeat domains, EF-hand motifs, a growth arrest specific 2 (GAS2)-related protein (GAR) domain and a domain containing a series of glycine–serine–arginine (GSR) repeats. Interestingly, of all the members of the plakin family, only plectin has been unambiguously shown to be expressed in astrocytes [192,193].

Nonetheless, a few reports indicate that desmoplakin might be present in human astrocytes in certain conditions, given that electron microscopy has revealed structures similar to desmosomes [194,195]. Desmosomes are a type of adhesive protein complex, specialized to form stable adhesive junctions between cells with which the N-terminal head domain of desmoplakin associates [196]. Similarly, astrocytes possess membrane structures that resemble hemidesmosomes (HDs) [195]. HDs are specialized integrin-mediated attachment structures that ensure the adherence of cells to the extracellular matrix by firmly anchoring IF networks [197]. Integrin α6β4, which is one of the two transmembrane components of mature (Type I) HDs, was identified in astrocytes [198]; however, BPAG1, which was initially discovered as a hemidesmosomal protein in keratinocytes [199], has thus far not been discovered in astrocytes, defining the astrocytic HD-like structures as Type II HDs. Considering that astrocytic morphological plasticity significantly depends on actin and tubulin [200], one would expect that MACF1, which interacts with microtubules and F-actin via distinct microtubule and actin-binding domains, is expressed in astrocytes. However, MACF1, although with confirmed expression in multiple tissues throughout the body [201], has only been identified in astrocytomas and glioblastomas, but not in normal brain tissue [202].

### 6.2. Plectin

The rat glioma cell line C6, in which plectin was first identified [203], is an experimental model system for the study of glioblastoma. Several years after the initial discovery, plectin expression was also confirmed in normal astrocytes [192,204] as well as in most other mammalian cell types [205,206]. IFs are one of the most important binding partners of plectin. Plectin was originally isolated as a major component of IFs extracted from cell lysates [203]. All plectin isoforms contain a high-affinity IF-binding site at their C termini, which mediates the targeting and anchorage of IFs at different, clearly defined cellular locations [207]. The remarkable isoform diversity of plectin is a consequence of differential splicing of 12 alternative first exons into a common exon 2, which is the first of seven exons (exons 2–8) encoding plectin’s ABD [207,208]; however, three of the alternative first exons are non-coding, giving rise to isoforms with truncated ABDs. The ABD of plectin is relatively well conserved and similar to the ABDs in spectrin, dystrophin, BPAG1 and MACF/ACF7 [209]; however, it is unique in presenting with two short exons (2α, 3α) that optionally splice into the ABD sequence, resulting in three different ABD isoforms [210]. The CH1 subdomain of plectin’s ABD contains another binding site for IFs, which likely favours the binding of soluble vimentin [210]. In general, the interaction of plectin with vimentin and lamin B was shown to be differentially regulated by protein kinase A and C [211,212]. In primary astroglial cells, a functional manifestation of plectin deficiency was reported regarding cAMP-dependent signaling pathways, because the cells showed a delay in cAMP-stimulated morphological differentiation [213]. Morphological (and functional) adaptations of astrocytes (i.e., reactive astrogliosis) are especially important as responses to CNS injury [101]. Considering the interdependence of plectin and IFs, it is not surprising that, in reactive astrocytes (specifically in the A2 subtype), the augmented expression of GFAP and vimentin is accompanied by the increased expression of plectin [193], as shown in Figure 2. Hence, it is reasonable to assume that plectin plays an important role in reactive astrogliosis. However, further research is needed to shed light on the mechanism and pecking order of IF and plectin action that leads to the resulting physiological response. Some parallels can be drawn from the involvement of plectin in Alexander disease, a rare progressive neurodegenerative disorder caused by dominantly acting mutations in GFAP. Rosenthal fibers are the hallmark of this disease (i.e., cytoplasmic proteinaceous aggregates in astrocytes [63], which, among other proteins, contain plectin and GFAP). Although the disease results in increased expression levels of plectin and GFAP in the brain, the proportion of both proteins appears to be relevant as well [64]. The overexpression of plectin cDNA converted these aggregates to networks composed of thin filaments, whereas the expression of GFAP with the most common Alexander disease mutation lowered plectin levels. These results suggest that insufficient amounts of plectin promote GFAP aggregation and the formation of Rosenthal fibers in Alexander disease [64]. However, the disease most frequently associated with plectin deficiency is epidermolysis bullosa simplex with muscular dystrophy (EBS-MD), a subtype of EBS. EBS, which happens to be the first disease identified involving IFs [214,215], is characterized by intraepidermal skin split (i.e., blistering of the skin). Mutations in the human plectin gene may result in autosomal recessive EBS-MD, EBS-MD with myasthenic features (EBS-MD-MyS), EBS with pyloric atresia (EBS-PA) limb girdle muscular dystrophy type 2Q (LGMD2Q), skin-only EBS [216] and the autosomal dominant variant EBS-Ogna [207,217].

The binding versatility of plectin is not limited to IFs, because plectin can associate with microtubules (directly or via microtubule-associated proteins) and actin filaments, the nuclear envelope, transmembrane receptors, proteins of the plasma membrane protein skeleton, mitochondria and signal transducers, such as kinases involved in migration and proliferation [205,213,218]. These interactions are mirrored by plectin’s multiple functions in practically all mammalian cell types [205,207]. However, considering the importance of IFs in astrocytes, coupled with the relatively limited amount of knowledge of cytolinker proteins and other cytoskeletal macromolecules in this cell type, there is no doubt that further research is warranted.

## 7. Conclusions

Astrocytes and IFs share humble beginnings in the literature, as studies of both were overshadowed by those performed on neurons and microtubules/actin filaments, respectively. The primary reason for the early absence of research interest in astrocytes and IFs is that, initially, both were perceived to provide solely mechanical support and were considered to be static in nature. In contrast, in the last few decades astrocytes have been shown to possess numerous functions in the CNS, the sum of which apparently exceeds those performed by neurons. Similarly, IFs have been demonstrated to be not only significantly more diverse, compared to microtubules/actin filaments, but to participate in a variety of dynamic processes, such as in cell signaling. 

Astrocytes are implicated to be actively involved in many CNS pathologies, either directly or indirectly. These pathologies result in the hypertrophy of astrocytic processes, a process that is known as reactive gliosis and encompasses a variety of biochemical, molecular, and morphologic events. The nature of reactive gliosis is highly heterogeneous and can, depending on the circumstances, either protect or perpetuate the underlying disease. A hallmark of reactive gliosis is the upregulation of certain IFs, namely of GFAP, vimentin, nestin and also, in some reactive astrocytes, synemin. Significant progress has been made in recent years in our understanding of the molecular mechanisms by which these IFs are orchestrating reactive gliosis. Considering that different IFs are interacting with each other and with various proteins, the whole IF scaffold is effectively implicated in practically all cellular processes. In order to fill the gaps in the current knowledge of individual IFs and their complex networks in astrocytes, both in physiological and pathological settings, a great deal of work remains to be done. In addition, two key challenges will be: (i) to assess the role of lamins in reactive gliosis, bearing in mind their capacity to regulate genes in a response to mechanical cues; (ii) to learn how the considerable complexity of cytolinker proteins, such as plectin and other cytoskeletal macromolecules, contribute to different stages of reactive gliosis. 

## Figures and Tables

**Table 1:** Astrocytes express five types of IF proteins. GFAP and vimentin isoforms are splice variants of a single gene and lamin isoforms are encoded by different genes.

IF Protein	Type	Isoforms in Astrocytes	Size (kDa)	Function in Astrocytes
Glial fibrillary acidic protein (GFAP)	III	α, β, γ, δ (ε), ξ, κ, Δ135, Δ164, Δexon6, Δexon7 [18,37]	38–50 [38]	Resistance to mechanical stress, cell migration and motility, mitosis, myelinization, maintenance and permeability of the blood–brain barrier, neurogenesis, chaperone-mediated autophagy, vesicle mobility, glial scar formation, response to hypoosmotic stress [12,13,32,37,41,42,43,44,45]
Vimentin	III	No splice variants reported in astrocytes	57 [46]	Mechanical integrity of cells and tissues, neurogenesis, glial scar formation, vesicle trafficking, cell morphology, cell motility, cell division, response to hypoosmotic stress [12,13,41,43,44,45,47,48,49]
Nestin	VI	No splice variants reported in astrocytes	240 [50]	Shape of protrusions, neurogenesis, cell motility [44,51]
Synemin	VI	α, β (human), H, M, L (mouse)	230 [52], 180 H (α),150 M (β), 41 L [53,54]	Astrocytoma motility [55]
Lamin	V	A, B1, B2, C	60–70 kDa [56]	Control of gene expression [57], transport of neurofibromin to the nucleus [58], nucleocytoplasmic transport [59]
